# Xander: employing a novel method for efficient gene-targeted metagenomic assembly

**DOI:** 10.1186/s40168-015-0093-6

**Published:** 2015-08-05

**Authors:** Qiong Wang, Jordan A. Fish, Mariah Gilman, Yanni Sun, C. Titus Brown, James M. Tiedje, James R. Cole

**Affiliations:** Center for Microbial Ecology, Michigan State University, East Lansing, MI USA; Department of Computer Science and Engineering, Michigan State University, East Lansing, MI USA; Department of Microbiology and Molecular Genetics, Michigan State University, East Lansing, MI USA; Department of Plant, Soil and Microbial Sciences, Michigan State University, East Lansing, MI USA

**Keywords:** Metagenomics, Assembly, Functional gene, HMM, Nitrogen cycle, *nifH*, *nirK*, Biofuel crop

## Abstract

**Background:**

Metagenomics can provide important insight into microbial communities. However, assembling metagenomic datasets has proven to be computationally challenging. Current methods often assemble only fragmented partial genes.

**Results:**

We present a novel method for targeting assembly of specific protein-coding genes. This method combines a de Bruijn graph, as used in standard assembly approaches, and a protein profile hidden Markov model (HMM) for the gene of interest, as used in standard annotation approaches. These are used to create a novel combined weighted assembly graph. Xander performs both assembly and annotation concomitantly using information incorporated in this graph. We demonstrate the utility of this approach by assembling contigs for one phylogenetic marker gene and for two functional marker genes, first on Human Microbiome Project (HMP)-defined community Illumina data and then on 21 rhizosphere soil metagenomic datasets from three different crops totaling over 800 Gbp of unassembled data. We compared our method to a recently published bulk metagenome assembly method and a recently published gene-targeted assembler and found our method produced more, longer, and higher quality gene sequences.

**Conclusion:**

Xander combines gene assignment with the rapid assembly of full-length or near full-length functional genes from metagenomic data without requiring bulk assembly or post-processing to find genes of interest. HMMs used for assembly can be tailored to the targeted genes, allowing flexibility to improve annotation over generic annotation pipelines. This method is implemented as open source software and is available at https://github.com/rdpstaff/Xander_assembler.

**Electronic supplementary material:**

The online version of this article (doi:10.1186/s40168-015-0093-6) contains supplementary material, which is available to authorized users.

## Background

Metagenomics faces scalability challenges stemming from the amount of raw sequencing data necessary to describe complex microbial communities, now often termed the microbiome [1, 2]. Metagenomic assembly has been an area of growing interest in the past decade, with early datasets assembled using single-genome assembly methods that had difficulty with metagenomic samples [3, 4]. The tendency for assemblers to only assemble dominant organisms while producing only fragmented partial assemblies for less dominant organisms, with limited recovery for any individual genes, has been an impetus to develop better metagenomic-specific assembly methods [5, 6].

We propose a gene-targeted assembly approach called Xander for assembling metagenomic datasets. Xander is a de Bruijn graph [7] assembler [8] that uses external information to perform a guided, instead of exhaustive, traversal of the assembly graph. Xander uses profile hidden Markov models (HMMs) [9] to guide graph traversal (HMM-guided assembly). An HMM can be considered as a directed probabilistic graph. Built from the alignment of a set of homologous sequences, an HMM quantifies position-specific conservations of the underlying sequence family. Using an HMM, the paths most likely to code for the target gene can be extended first thus limiting the portion of the assembly graph that must be explored. In addition to limiting the graph traversal, the HMM provides a measure of how likely the resulting assembled contig comes from the supplied model and, by inference, whether the contig actually codes for the gene of interest. Xander enables researchers to target specific genes involved in biologically interesting pathways without using amplicon-based sequencing approaches that are prone to primer limitations and PCR artifacts and that produce sequences too short to more fully characterize the genes.

Gene-targeted assembly is less resource intensive and faster than traditional whole-genome metagenomic assembly. In addition to the de Bruijn graph, only small paths relative to the graph’s size must be kept in memory. In our initial implementation, further reductions in memory usage are achieved by using a probabilistic data structure for holding the graph in memory, a Bloom filter [10–12]. By targeting relatively small segments of the assembly graph using an HMM to guide assembly, the amount of the graph that must be explored during assembly is constrained, providing a speedup over whole-genome approaches.

The targeted assembly of metagenomic datasets has drawn research interest, generating several approaches including EMIRGE, Mira, and SAT-Assembler [13–15]. These targeted methods first identify reads likely to be from targeted genes before assembly, as does the reference-assisted assembler IDBA-Hybrid [16]. Instead, Xander begins with a de Bruijn graph representation based on all reads, as most current bulk assemblers do. A de Bruijn graph approach has also been used for a BLAST-like search algorithm, BlastGraph [17]. BlastGraph uses a graph representation of a reference database that can then be queried with unknowns, whereas Xander queries the graph representation of metagenomic reads using HMMs. Other work used an HMM to search sequences in an indexed tree structure [18], but only short HMMs up to length 12 were tested.

We validated our method using defined community metagenomic data and then applied the tool to 21 rhizosphere soil samples totaling 800 giga base pairs (Gbp) from three different biofuel crops. We targeted three genes: one conserved taxonomic gene and two genes from different parts of the nitrogen cycle that are both critical to plant productivity.

## Implementation

### Xander graph structure

**Fig. 1:**
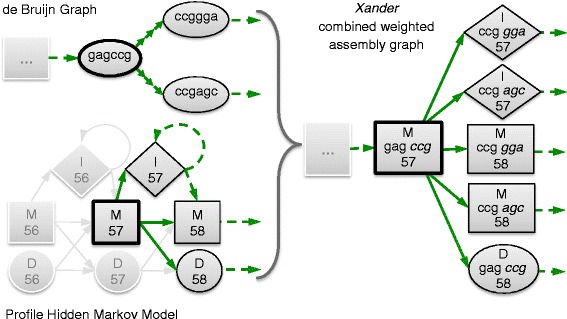
The Xander combined weighted assembly graph structure. *M*, *I*, and *D* represent HMM match, insert, and delete states, respectively. *Numbers* represent state position on the HMM. For simplicity, a kmer length of 6 is used and weights of the edges are not shown. The vertices shown in *bold* on the de Bruijn graph and profile hidden Markov model are combined to form the *bold vertex* in the combined graph. The *green solid arrows* represent all possible outgoing edges from these vertices. *Boxes with ellipses* indicate additional omitted graph structure. The delete HMM state is combined with the de Bruijn graph vertex from the last match; this carries forward the state information necessary to correctly form subsequent vertices in the combined graph. During path search, if this combined vertex becomes the best scoring vertex in the open set, it is removed from the open set and the adjacent combined vertices are instantiated and added to the open set

Xander requires two sets of input sequences: a set of reference sequences of the targeted genes to build a protein profile HMM and one or more metagenomic read files to build a de Bruijn graph (DG). An HMM can be considered as a directed probabilistic graph with transition and emission probabilities between states. A novel graph structure was created to combine the DG and HMM together into a single combined weighted assembly graph (CAG) (Fig. 1).

For a graph G, let *V*(G) and *E*(G) be the vertex set and edge set, respectively. A vertex *w* in CAG is created for every pair of vertices *u* and *v*, where *u* belongs to *V*(DG) and *v* to *V*(HMM). The total number of vertices in CAG will be |*V*(DG)| * |*V*(HMM)|. The edge $\overrightarrow{w_i{w}_j}$ between vertices *w*_*i*_ and *w*_*j*_ in the edge set *E*(CAG) is made by combining vertices *v*_*i*_ with *u*_*i*_ and *v*_*j*_ with *u*_*j*_, respectively:

$\overrightarrow{w_i{w}_j\ }\in\ E\left(\mathrm{C}\mathrm{A}\mathrm{G}\right)\ \leftrightarrow\ \overrightarrow{v_i{v}_j\ }\in\ E\left(\mathrm{H}\mathrm{M}\mathrm{M}\right)\kern0.5em \mathrm{and}\kern0.5em \overrightarrow{u_i{u}_j}\ \in\ E(DG)$ if *v*_*j*_ is an insert or match state.

$\overrightarrow{w_i{w}_j\ }\in\ E\left(\mathrm{C}\mathrm{A}\mathrm{G}\right)\leftrightarrow\ \overrightarrow{v_i{v}_j\ }\in\ E\left(\mathrm{H}\mathrm{M}\mathrm{M}\right)\kern0.5em \mathrm{and}\ {u}_i={u}_j$ if *v*_*j*_ is a delete state.

The weight of an edge $\overrightarrow{w_i{w}_j}$ in CAG is defined as the log of the product of the transition and emission probabilities taken from the HMM:

$\mathrm{weight}\left(\overrightarrow{w_i{w}_j}\right)$ = log [ℙ_transition_(*v*_*i*_→*v*_*j*_)] + log [ℙ_emission_(*v*_*j*_)] if *v*_*j*_ is a match state, $\mathrm{weight}\left(\ \overrightarrow{w_i{w}_j}\ \right)\kern0.5em =\kern0.5em  \log \left[{\mathrm{\mathbb{P}}}_{\mathrm{transition}}\left({v}_i\ \to\ {v}_j\right)\right]$ otherwise.

The emission symbol is the terminal character(s) in the substring of length k (kmer) contained in *u*. The underlying DG graph is constructed in nucleotide space regardless of whether the HMM is modeling protein or nucleotide sequences. When searching with a protein HMM, the DG graph is converted to protein space by walking three vertices in any one direction at a time. The emission symbol then becomes the last three nucleotide characters of the kmer translated to a single amino acid (aa). The vertex chosen to begin graph traversal thus determines the translation reading frame.

By default, Xander builds a graph using all the kmers from the reads regardless of the abundance of the kmers. Xander implements a counting Bloom filter to store the counts of each kmer. This allows optionally filtering out kmers failing to meet the minimum abundance cutoff during the graph-building step.

#### Assembly approach

Xander assembly starts at a vertex corresponding to a kmer contained in the target gene. Since starting vertices can be in any model position, not just the beginning of the model, a second HMM is built from the reverse of the seed alignment used to build the forward HMM. Using this reverse model, Xander can traverse paths in both directions from a starting vertex. From a starting vertex, Xander searches for the best path through the CAG to a goal vertex, a vertex corresponding to one end of the HMM. The contigs generated by each search direction are output separately and then combined into a single contig.

To identify starting kmers, Xander uses a set of aligned reference sequences from the target genes and the read files. The reference set used at this step can be larger than the one used to build HMMs and can include partial and lower quality sequences to provide broader coverage of the organisms. All overlapping kmers from the reference sequences are stored in a hash table. Each read is decomposed into kmers to search for matches in the hash. If a perfect match is found, the kmer, alignment position, and corresponding implied HMM match states form a starting vertex for the next assembly step. For use with a protein HMM, a peptide kmer length of one third of the input kmer size is used and input reads are translated into all six reading frames. When assembling multiple target gene families, the reference sets can be combined together into a single hash so that search starts can be identified in a single pass over the reads.

To assemble contigs, Xander searches from the starting vertices corresponding to the identified starting kmers using the A* search algorithm [19] to find the best paths through the CAG. The set of goal vertices is defined as any vertex constructed from the terminal HMM model position match or delete state. The scoring function for a path *P* is defined as follows: $S(P)={\displaystyle {\sum}_{i=1}^{\left|P\right|}}\mathrm{weight}\left(\overrightarrow{w_{i-1}{w}_i}\right)$ where the *weight* function returns the weight of the edge between two vertices in *P*.

The A* algorithm maintains an “open set” of examined partial paths from the starting kmer ending in a vertex *w*. The open set is sorted by the expected score for the best path through *w* to a goal vertex, which is equal to the sum of the score of the partial path to *w* and of the score from *w* to a goal vertex using a heuristic cost function *h*(*w*). Let vertex *v* be the HMM component of *w* and *m*_*i*_ be the match state HMM vertex following *v. h*(*w*) is defined as follows:

$$ h(w)= \log \left[\mathrm{\mathbb{P}}\left(v\to {m}_i\right)\right]+{\displaystyle \sum_i^{L-1}} \log \left[\mathrm{\mathbb{P}},\left({m}_i,\to, {m}_{i+1}\right)\right] $$

That is, the sum of the most likely state transitions from *v* to the end of the model, where ℙ is the probability of the given transition and emission of the most likely amino acid, and *L* is the length of the HMM. Since this is the best scoring path from *v* to the goal for any possible sequence, it meets the admissibility criteria for A*.

The emission probabilities produced by HMMER are normalized to a null model, meaning that some normalized emission values are greater than one. To ensure the log-odds edge weights used by the heuristic score and the scoring function were both monotonic, the log of the maximum normalized match emission probability was subtracted from the edge weights for the preceding match → match, insert → match, match → delete, delete → match, and delete → delete edges. These modified weights are used except where we explicitly refer to the unmodified weights below.

The A* algorithm proceeds by successively examining the best scoring vertices from the open set. If a best scoring vertex *w* is not a goal vertex, the adjacent vertices are added to the open set and *w* is removed from the open set and added to a closed set. We cache the best paths found from previous searches to speed up subsequent searches. If *w* is on a cached shortest path, we only open the path to the next vertex on the cached best path.

A path-pruning heuristic modification to the A* search is implemented in Xander to terminate paths that are unlikely to yield contigs that match the model well. In addition to the path score calculated from the monotonic (modified) edge weights, a standard *HMM score* is calculated from the unmodified weights and maintained for each partial path, along with the position of the maximum HMM scoring vertex *w*_*m*_ in the path to *w*. When a vertex *w* reaches the top of the open set, it is discarded (pruned) if the HMM score along the path to *w* has not improved within a user-specified number of vertices. In the event a search terminates (the open set is empty) before reaching the end of the model, the path to *w*_*m*_, the intermediate vertex with the highest HMM score is returned.

The A* algorithm only finds one single shortest path from a starting vertex. To explore microheterogeneity, Xander implements an option to find multiple high-scoring paths from a single starting vertex using Yen’s K shortest path algorithm [20]. This algorithm iteratively finds the shortest path then the second shortest path to the *k*th shortest path. This is sped up by the observation that the *i*th shortest path in the sequence must branch from one of the *i* − 1 shortest paths already identified. Yen’s algorithm can be further improved by the observation that the *i*th shortest path must branch from its parent *j* after the point *j* branched from its parent [21]. In Xander, we have modified Yen’s algorithm to find the next best path *P*_*i*_ containing at least one edge not present in the previously found *i* − 1 best paths. This avoids a combinatorial explosion of best paths from a small number of minor variations. A contig sequence may still be common for multiple paths, representing variations in the alignment between the HMM and the sequence, so additionally, the implementation does not return paths that do not contain any previously unseen kmers. In this way, each of the K paths returned contains new sequence information.

#### HMP-defined community data

The Human Microbiome Project (HMP)**-**defined community consists of 22 human-gut-associated microorganisms (Additional file 1: Table S1). Only the 20 bacterial organisms were used in this analysis. The reference genomes were downloaded [22], and the annotations for each organism were downloaded from GenBank. Two whole-genome shotgun sequence datasets for the HMP-defined community were downloaded from NCBI’s Short Read Archive (SRR172902, SRR172903). The two datasets were combined for analysis in this study. The combined set consists of a total of 1037 Mbp of length 75 bp Illumina reads. Quality filtering was performed by trimming reads at a quality score of “B” as recommended by Illumina (CASAVA 1.7 User Guide) using the RDPTools ReadSeq package [23].

#### Rhizosphere soil data

Metagenomic sequence was produced by the Joint Genome Institute (JGI) as part of the Great Lakes Bioenergy Research Center’s (GLBRC) sustainability research theme. The samples derive from rhizosphere soil collected at Kellogg Biological Station intensive sites [24] in October 2012 from three biofuel crops: *Zea mays* (corn), *Miscanthus × giganteus* (*Miscanthus*), and *Panicum virgatum* (switchgrass). For each crop, there are seven biological replicates (Additional file 1: Table S2). Sequence reads and metadata are available from JGI [25]. Each read file contains paired-end reads of length 150 bp from one lane of Illumina HiSeq, one file, or lane, per rhizosphere sample. We downloaded the bulk assembly contigs (minimum length 300 bp) from MG-RAST [26]. In brief, these bulk assemblies were created by first merging the paired-end reads, then all seven replicates of each crop were pooled together before assembly using the diginorm/partitioning assembly pipeline [27] (see Additional file 1). To be consistent with the bulk assembly, we used the merged reads as the starting data for Xander assembly.

#### HMM construction

HMMs were built using the “seed sequences” for the corresponding genes from RDP’s FunGene site [23]. These seed sequences were used to build a forward HMM and a reverse HMM for each gene using a modified version of HMMER3 [28]. Since HMMER3’s default settings are tuned for detecting remote paralogs [29] whereas Xander is targeting close homologs, we used the “-- enone” option to disable sequence weighting. The default priors sometimes caused extensive searching of nonproductive insert and delete paths. HMMER3’s source code was modified to change the prior probabilities for the *delete → match* and *insert → match* transitions to 95 % probability and *delete → delete* and *insert → insert* transitions to 5 % probability. The modifications to HMMER3 are available as a patch file against version 3.0.

#### Reference sets

From FunGene release version 7.5.3, we downloaded a set of 263 unique near full-length nitrite reductase (*NirK*) protein and corresponding nucleotide sequences using the following filters: minimum aa 300; minimum HMM coverage 80; and minimum HMM score 300. The average length of the protein sequences was 410 aa. A set of 1734 near full-length ribosomal protein L2 (*RplB*) protein and corresponding nucleotide reference sequences (average length of 280 aa) was selected from the same site with these filters: minimum aa: 250; minimum HMM coverage 90; and minimum HMM score of 440. For nitrogenase reductase (*NifH*), we used the set of 782 near full-length reference sequences with average length of 300 aa that was used as references for the FrameBot tool in a previous publication [30]. These protein reference sets were used with Xander to identify starting kmers and by FrameBot to find the closest matches to the contigs. The nucleotide reference sets were used with UCHIME [31] to detect chimeras. All these reference sets are available from the Xander_assembler package (see Availability and requirements below).

#### Bulk assembly of rhizosphere soil data

Bulk assemblies were provided by Jiarong Guo. The following section briefly describes the bulk assembly steps [Jiarong Guo, personal communication] for the 21 rhizosphere soil samples deposited in MG-RAST [26]. The raw reads were trimmed starting at bases with the quality score of “#” near the distal end and the paired-end reads were merged using Flash [32] with parameters “-m 10 -M 120 -x 0.20 -r 140 -f 250 -s 25 -t 1.” The sizes of the merged files in FASTA format range from 27 to 57 gigabytes (GB) (Additional file 1: Table S2). The merged reads from all seven replicates of each crop were pooled together before bulk assembly using the diginorm/partitioning assembly pipeline [27]. The minimum length of contigs is 300 bp. The reads were mapped to the contigs using BWA [33].

#### Targeted gene identification in bulk assembly

Protein sequences were translated from the bulk assembly contigs downloaded from MG-RAST using FragGeneScan [34]. We used HMMER3 to search for NirK, NifH, and RplB from these protein sequences using the forward HMM models used by Xander. Only hits with a minimum HMM score of 50 were retained. Hits were clustered at 99 % aa identity. The closest matches to the representatives were identified using the RDP AlignmentTool package [23] against the protein reference sets.

#### Xander processing steps for rhizosphere data

**Fig. 2:**
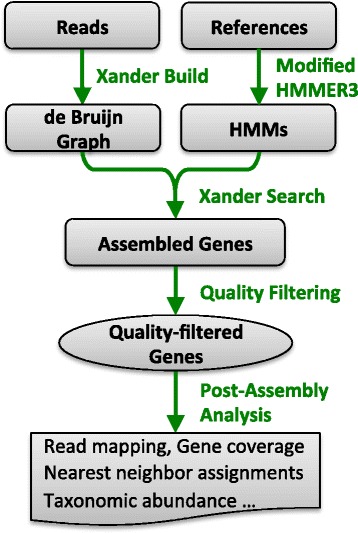
Xander gene assembly workflow. Two types of input sequences are required: one or more metagenomic read files used to build the de Bruijn graph and one set of reference sequences for each targeted gene, for building specialized profile HMMs using a modified version of HMMER 3.0 (see the “Implementation” section). During the search phase, Xander uses a combined weighted assembly graph to assemble genes (contigs). After assembly, several filters are applied at the quality filter step: chimeric genes, or genes below length cutoff or HMM score cutoff are discarded, and genes are clustered at 99 % aa identity and the longest one from each cluster is chosen as the representative. The quality-filtered genes are further processed to provide coverage and abundance information

For each of the three genes and for each sample (either individual or pooled), we used Xander to assemble one best contig from each starting kmer of length 45. To be comparable to the metagenomic assembly contigs, assembled contigs shorter than 300 nucleotides or with an HMM score less than 50 were discarded. A few post-assembly steps were included as part of the analysis (Fig. 2). We clustered the assembled contigs at 99 % aa identity and chose a set of representative nucleotide and protein contigs (the longest contig from each cluster). The 99 % aa identity cutoff was used for contig clustering throughout the analysis unless otherwise noted. Chimeras were identified using UCHIME against the nucleotide reference set. The closest matching reference sequences to these representative contigs were identified using FrameBot. Read mapping and kmer coverage estimates were also performed as described below.

#### Gene abundance and kmer coverage estimate

We used the single copy core *rplB* gene to normalize gene abundance. The relative abundance for a particular gene, e.g., *nirK*, was estimated as the ratio of the number of reads covering at least one kmer in the *nirK* gene contigs divided by the number of reads covering at least one kmer in the *rplB* gene contigs. The same kmer length used for the assembly step was used to estimate coverage.

The mean nucleotide kmer coverage for each representative nucleotide contig was calculated from the reads using a tool included in the Xander package. When a read had a kmer present in multiple contigs, the counts for that kmer were equally divided among the contigs to avoid overcounting. The kmer coverage of a contig is then calculated as the mean counts of all the kmers included in that contig.

#### Ordination analysis

For each of the *nirK* and *rplB* genes, the representative protein contigs from each of the 21 rhizosphere soil samples were aligned using HMMER3 and all samples clustered together using RDP mcClust [23] with the complete linkage algorithm. The operational taxonomic unit (OTU) abundances at 95 % aa identity were corrected using the mean nucleotide read coverage of each contig before principal component analysis (PCA). Vegan package version 2.0-3 in R 2.15 was used to perform PCA.

#### SAT-Assembler processing

SAT-Assembler [15] was run with the default settings. The HMMs were created with the unmodified HMMER 3.0, with the seed sequences used for creating Xander-specific HMMs.

#### Computer resources

The MSU High Performance Computing Center (HPCC) [35] cluster was used for the majority of experiments. SAT-Assembler failed to complete assembly of the HMP + corn 1 dataset after 7 days, the maximum allowed runtime on the HPCC (Xander completed all steps in 12 h). We assembled these data with SAT-Assembler by using a faster machine (iMac with 3.2-GHz Intel Core i5 processor with local drive) in 124 h.

## Results

### HMP-defined community analysis

We used the HMP-defined community data to evaluate Xander’s performance with *rplB* genes selected as the initial assembly targets. The *rplB* gene was selected because it is a well-conserved single-copy core gene. Each defined community organism has a single copy of the *rplB* gene, and the gene sequences of several members share identical kmer of length 30 or greater. The average length of the *rplB* genes from the defined community organisms is 825 bp.

We evaluated Xander assembly quality by tuning three parameters: kmer length, minimum kmer cutoff, and prune cutoff. Since none of the *rplB* sequences from the defined community members are covered by kmers of length 60, we tested kmer lengths of 30 and 45. For kmer lengths of 30 and 45, we built de Bruijn graphs with a minimum kmer cutoff 1 (count 1 defined community graph) or 2 (count 2 defined community graph). We then searched the defined community graph using all the starting kmers identified in the reads using the 20 bacterial members of the defined community (Additional file 1: Table S1) as a reference set. We measured the number of differences between the nucleotide contigs and the *rplB* genes from the defined community members. When a kmer length of 30 was used, Xander was able to assemble some full-length contigs that match with no or few differences to the defined community members but produced some chimeric contigs between defined community members (see Additional file 1). We found that using a larger size kmer of 45 reduced the number of chimeras returned from 30 % with length 30 to 0.3 % (one partial-length sequence).

The sets of unique contigs returned were identical to the results without heuristic pruning for values of prune of 5, 10, 15, and 20, except the count 1 defined community graph search with prune 5 returned one sequence 21 bases shorter at the 3′ end. In all cases, decreasing the value of the prune decreased the total number of vertices opened, although the relative savings were greater for the kmer length of 45 (Table 1). The number of vertices opened using the length 45 count 1 defined community graph with prune 20 was only 10.7 % of the number opened without pruning. It is worth mentioning that the number of vertices opened without pruning using kmer length of 45 is only 0.76 % of the ones opened using kmer length of 30 with count 1 defined community graphs.

**Table 1:** Percent of vertices opened with pruning compared to no pruning with the corresponding length and count

Prune cutoff/kmer length	Length 30		Length 45	
	Count 1^a^	Count 2^b^	Count 1^a^	Count 2^b^
No pruning (# opened * 10^6^)	2325	55.8	17.7	9.2
Prune 5 (% opened)	1.55	16.53	NA	NA
Prune 10 (% opened)	2.66	25.09	NA	NA
Prune 15 (% opened)	6.34	29.28	NA	NA
Prune 20 (% opened)	11.58	31.86	10.7	6.5

^a^Count 1: de Bruijn graph requiring kmers with minimum abundance of 1 in the reads

^b^Count 2: de Bruijn graph requiring kmers with minimum abundance of 2 in the reads

Xander can produce multiple paths from one starting kmer using a modified Yen’s K shortest path algorithm. To explore multiple paths, we used the de Bruijn graph of kmer length of 45 to search the best 1000 paths from one starting kmer that produced a contig perfectly matching *Staphylococcus epidermidis*. Xander assembled 176 unique nucleotide contigs. These contigs formed six clusters at 99 % aa identity. No chimeric sequences were found. The representative nucleotide contigs shared 98.5 to 99.9 % nucleotide identity to *S. epidermidis*.

### Mixing HMP and corn rhizosphere data

We tested how well Xander assembly worked when the HMP-defined community data were mixed with reads from rhizosphere sample C1 (corn replicate no. 1). We built a de Bruijn graph of size 33 GB using kmer length of 45 and minimum count of 1. We searched this graph for *rplB* using the starting kmers from the HMP-defined community and assembled 79 unique contigs. Six contigs were chimeras with parents related to *Staphylococcus aureus* and *Bacillus megaterium*, *Enterobacter sp*. and *Serratia marcescens*, or *Deinococcusradiodurans* with an unknown second parent (this latter chimera was obvious from inspection). The remaining contigs formed 19 clusters at 99 % aa identity. Compared to the results using the defined community reads alone, Xander assembled a representative full-length contig perfectly matching *S. epidermidis*. Xander also assembled one partial representative contig matching *Streptococcus mutans* and one matching *S. aureus* with 1 and 2 mismatches, respectively. In addition, although we only searched starting from kmers in the HMP-defined community, Xander assembled contigs matching *rplB* reference sequences not present in the defined community: one full-length contig sharing 99 % nucleotide identity with *B. megaterium*, one full-length contig, and one partial contig sharing 99 % nucleotide identity with *Enterobacter cloacae*.

We explored the effect of kmer abundance filtering by using minimum kmer count 2 and kmer length 45 with the mixed datasets. None of the defined community members have all kmers present in the count 2 graph. Xander assembled 23 unique contigs. There were 17 clusters with three representatives identified as chimeras. Compared to the count 1 graph, the contig matching *S. epidermidis* was 102 bases shorter, whereas the contig matching *S. aureus* was full length and 210 bases longer than the one assembled by using the count 1 graph. One contig from the count 1 graph matched *D. radiodurans*, but none from the count 2 graph matched. The rest of the contigs were actually closer to *rplB* reference sequences not present in the defined community. The average length of the assembled contigs was very similar between the count 1 and count 2 graphs.

When searching the best 1000 paths using the same starting kmer of length 45 used in the defined community reads alone, Xander assembled the same set of contigs as those using the defined community reads alone.

### Comparison to the gene-targeted SAT-Assembler

When we tested the SAT-Assembler on the HMP-defined community data, SAT assembled 798 *rplB* contigs averaging 96 bp in length. Only four unique nucleotide *rplB* contigs were longer than the 450-bp cutoff used with Xander (see Additional file 1). These four contigs shared a median 97.8 % nucleotide identity to three defined community members (Table 2). Two of the contigs (588 and 512 bp) were chimeric, the remaining two (533 and 559 bp) shared 99.6 and 99.8 % nucleotide identity to *Bacteroides vulgatus*, respectively. All four contigs were missing both ends of the genes. Using prune 20 and kmer length of 45, Xander assembled six full- or near full-length (median coverage 94.6 %) contigs matching four defined community members with a median 99.8 % nucleotide identity to the closest references. At kmer length of 45, Xander did not assemble contigs matching *B. vulgatus*. At kmer length of 30, Xander assembled a *B. vulgatus* contig of length 681 bp with one mismatch.

**Table 2:** Comparison between Xander and SAT assembly of ribosomal protein L2 (*rplB*) genes from HMP-defined community data

Tool	SAT	Xander^a^
# contigs	4	6
# members covered	3	4
Median gene coverage^b^ (%)	75.7	94.6
Max gene coverage^b^ (%)	79.9	100
Median % nucleotide identity	97.8	99.8
Max % nucleotide identity	99.8	100

^a^Xander: kmer length of 45, prune 20, count 1 graph

^b^Gene coverage: length of the contigs compared to the closest defined community members

We also attempted to assemble HMP community member *rplB* genes from the mixed HMP and corn rhizosphere sample 1 data. SAT assembled 1353 contigs before clustering with length longer than 450 bp (median length 594 bp). Only three contigs shared more than 90 % identity with any defined community member. Two of these contigs of lengths 808 and 816 bp were near identical and appear to be chimeras between *B. vulgatus* on the 5′ end and an unknown organism for the last 190 bases of the 3′ end. A third short contig of 490 bp appears also to be chimeric, with the 5′ 350 bp 99 % identical to the corresponding region of the *D. radiodurans* sequence, and the final 140 bp only 67 % identical to *D. radiodurans*. In all three cases, we were unable to identify the other parent.

### Bulk assembly of pooled rhizosphere soil data

There were fewer than 50 *nirK* representatives found from each of the replicate samples pooled by crops, with sizes of 233 to 293 Gbp (Table 3). None of the *nirK* contigs were close to full length and shared only a median of 73.3 to 79.6 % aa identity with reference sequences. Corn samples showed slightly higher species richness (37 OTUs at 90 % aa identity) than the 30 in switchgrass and *Miscanthus* samples. There are 125 *rplB* clusters (420 reads mapped) found in corn, 166 clusters (487 reads mapped) in *Miscanthus*, and 146 clusters (354 reads mapped) in switchgrass samples. The estimated *nirK* gene abundances were between 25 and 30 %. More than half the *nirK* and *rplB* contigs had only median reads coverage of one or two (data not shown). Only two partial-length *nifH* genes were found in corn, three from *Miscanthus*, and one from switchgrass samples.

**Table 3:** Nitrite reductase (*nirK*) genes found in bulk assembly of pooled rhizosphere samples

Sample	Corn	*Miscanthus*	Switchgrass
File size (GB)	349	325	277
Data size (Gbp)	293	275	233
# protein contig clusters^a^	41	37	39
# OTUs at 95 % aa identity	38	33	34
Median length (aa)	131	115	130
Max length (aa)	234	252	301
Median % aa identity^b^	75.6	79.6	73.3
Max % aa identity^b^	95.1	94.3	92.1
# reads covering kmers	105	123	106
Gene abundance	0.25	0.25	0.3

^a^Number of protein contig clusters at 99 % aa identity

^b^Percent identity to nearest reference sequence

### Xander assembly of pooled rhizosphere soil data

Since the longer kmer and pruning performed better based on the results using the HMP-defined community data, a kmer length of 45 and prune 20 was used throughout the analyses described below. To be comparable with the bulk metagenomic assemblies, we used the same three pooled samples and used Xander to assemble *nirK*, *nifH*, and *rplB* genes in a single path search. Xander assembled at least 40 times more *nirK* contig clusters (Table 4) than the ones found by metagenomic assembly. The median length of these *nirK* contigs was 80 aa longer than the median length found in metagenomic assembly. They shared a median of 84.7 to 88.3 % aa identity with the references, higher than 73.3 to 79.6 % aa identity for the metagenomic assembly. Corn samples showed slightly higher species richness (413 OTUs at 90 % aa identity) than 358 in switchgrass and 317 in *Miscanthus* samples. The number of *rplB* contigs assembled was similar among the three crops. The majority of *rplB* contigs were near full length with a median length of 274 aa (compared to the average length of 280 aa for the reference sequences). Very few *nifH* genes were assembled.

**Table 4:** Xander assembly of pooled rhizosphere samples

Gene	*nirK*			*nifH*			*rplB*		
Crop	C	M	S	C	M	S	C	M	S
# chimeric clusters removed	16	207	11	0	1	0	14	28	44
# protein contig clusters^a^	1993	1807	1581	39	57	41	19,287	20,463	17,334
# OTUs at 95 % aa identity	741	674	582	14	24	17	6100	6887	6004
Median (aa)	215	230	208	294	256	255	274	274	274
Longest (aa)	380	372	370	296	296	296	285	285	284
Median % aa identity	88.3	84.7	87.8	92.7	91.9	91.6	77.7	75.8	76.3
Max % aa identity	100	99.4	98.6	100	100	100	100	100	100
# reads covering kmers	27,404	19,815	16,661	411	534	461	225,985	179,867	149,661
Relative abundance	0.121	0.11	0.111	0.002	0.003	0.003	1	1	1

*C* corn, *M Miscanthus*, *S* switchgrass, *nirK* nitrite reductase gene, *nifH* nitrogenase reductase gene, *rplB* ribosomal protein L2 gene

^a^# protein contig clusters: number of protein contigs clustered at 99 % aa identity

**Fig. 3:**
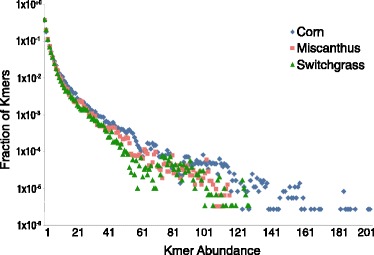
Kmer abundance of nitrite reductase gene (*nirK*) representative contigs assembled by Xander from the pooled rhizosphere samples. The representative contigs were chosen from clusters at 99 % aa identity. *X-axis* indicates the number of times (abundance) a kmer in the contigs occurred in the reads. *Y-axis* represents the fraction of total unique kmers with this abundance

We examined the kmer abundance and mean kmer coverage for each representative *nirK* and *rplB* contigs. More than half the kmers in the three samples occurred only once or twice. The corn sample had more high-coverage kmers than *Miscanthus* or switchgrass (Fig. 3, Additional file 1: Fig. S1). The corn sample also had more contigs with higher mean coverage than *Miscanthus* and switchgrass (Additional file 1: Fig. S2). Using the pooled samples, we estimated about 10 % of the organisms had *nirK* genes in these soil samples and only about 1 in 200 to 300 had *nifH* genes. These estimates were very similar between the three crops, and close to those obtained from one sample alone, but lower than those estimated by the bulk assembly (Table 3).

Among the pooled samples, about 15 % of the nirK contigs were the closest match to *rplB* from *Bradyrhizobium japonicum* USDA 110. We explored searching 1000 best paths from the pooled corn samples using one 3′ end starting *nirK* kmer from *B. japonicum* USDA 110. Xander returned 979 unique near full-length nucleotide contigs that formed 159 clusters at 99 % aa identity. The 159 cluster representatives shared 80.5 to 94.8 % aa identity with *B. japonicum* USDA 110 and four other *Bradyrhizobium* species*.* The contig sharing the highest percent aa identity with *B. japonicum* USDA 110 was not the best path but the 247th best path, with a very similar HMM score to the best path (1066.75 vs 1069.65).

### Xander assembly of individual rhizosphere soil samples

Using a 33-GB de Bruijn graph built for each of the 21 samples, Xander assembled, on average, 327 *nirK* contig clusters, with median length of 200 aa and median percent aa identity of 89.7 % to known *nirK* genes (Additional file 1: Tables S3, S4, and S5). Xander assembled 3957 *rplB* contig clusters with a median length of 266 aa (recall the average length of *rplB* references is 280 aa). Even though only a few *nifH* contigs were assembled for each sample, the median percent aa identity to known *nifH* genes is 94.8 %. Using kmer coverage to calculate the relative abundance, we estimated about 10 % organisms have *nirK* genes in these 21 soil samples (Additional file 1: Tables S3, S4, and S5). The average abundances of *nifH* genes in corn, switchgrass, and *Miscanthus* are 0.11, 0.22, and 0.24 %, respectively.

**Fig. 4:**
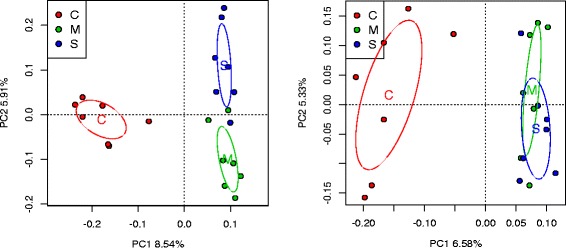
Principal component analysis of rhizosphere samples (*n* = 7 per crop). The OTU abundances at 95 % aa identity were corrected using the mean kmer coverage of each contig. The OTU data were then standardized using the Wisconsin square root normalization as implemented in R. *Ellipses* represent 1 standard deviation of the points from the centroid. *C* corn, *M Miscanthus*, *S* switchgrass. *Left*: nitrite reductase (*nirK*). *Right*: ribosomal protein L2 (*rplB*)

We used PCA to visualize the community structures among the 21 rhizosphere samples using either *nirK* or *rplB* representative contigs. The *nifH* contigs were not used due to the low number of contigs assembled. Both PCA plots showed that corn samples are distinct from the switchgrass and *Miscanthus* samples (Fig. 4).

### Xander performance

The Xander program contains three main steps. The graph-building step is single-threaded in our current implementation. The step to find starting kmers can be done in multiple threads and is easily parallelizable. Once the starting kmers are identified, the assembly of multiple genes can run in parallel. We choose three datasets with different data sizes to illustrate the memory usage and running time (Table 5). For example, the rhizosphere sample C1 required 60-GB memory and completed within 20 h on MSU’s HPCC using a single CPU. When tested on HPCC, the HMP-defined community data assembled in 25 min, while on an iMac with 3.2-GHz Intel Core i5 processor, it took 5 min, both single-threaded. Speed is expected to be faster when files are stored on a local drive.

**Table 5:** Xander processing statistics with kmer length of 45 and count 1

Sample name	HMP^a^	C1	Corn
File size (GB)	1.7	46	349
Build memory (GB)	1	60	200
Build time (h)	0.3	6.4	41
Find starting kmers (h)^b^	0.1	3.6	27.0
Search *nifH* time^c^	0.3 (min)	1 (min)	6 (min)
Search *nirK* time^c^	NA	48 (min)	36.7 (h)
Search *rplB* time^c^	1.1 (min)	228 (min)	49.4 (h)

^a^HMP-defined community data

^b^For single thread. Can be multi-threaded or run in parallel

^c^For single thread. Can be run in parallel

## Discussion

Xander uses a novel data structure combining de Bruijn graphs and HMMs to target assembly of important genes from metagenomic data. Using this data structure allows us to apply powerful graph search techniques to assemble individual genes. The HMM-guided assembly reduces the search space and provides gene annotation. Additional optimizations including caching of best paths from previous searches and heuristic search pruning provide several orders of magnitude of speedup over a naïve approach. Xander requires reference sequences from the targeted gene families for HMM training and start finding and is not meant for discovering completely novel gene families. Although we applied this method to soil rhizosphere metagenomic data, this method should also be applicable to *de novo* assembly of transcriptome and metatranscriptome data.

We targeted three gene families: *rplB*, a single-copy core phylogenetic marker gene; *nifH*, a marker for nitrogen fixation; and *nirK*, a marker for denitrification. There is considerable interest in using biofuel crops as one way to mitigate global warming. One factor in improving lower cost biofuel crop production on non-food-producing lands is to improve plant-available nitrogen. The Kellogg Biological Station has replicate field plots of corn, a traditionally farmed annual biofuel crop, and two perennial biofuel crops, an exotic, *Miscanthus*, and a native, switchgrass. We used Xander to assemble the three genes from the pooled rhizosphere samples from each crop. Roughly 17,000 to 20,000 *rplB* genes assembled for each sample (Table 4). We found about 10 % as many *nirK* contig clusters and even fewer *nifH* contig clusters. A previous study at other Midwest sites found higher gene richness in nitrous oxide reductase (*nosZ*), another marker gene for denitrification, in corn soil communities than in switchgrass and *Miscanthus* soil communities [36], as did we for *nirK*. They also found lower abundance of *nifH* compared to *nosZ* in all soils, matching our findings. When compared to the available bulk metagenomic assembly using the diginorm/partitioning assembly pipeline used in several recent published works [12, 27], only 125 to 146 *rplB* gene clusters were found in the available bulk metagenomic assembly (Table 3). In general, Xander assembled 10- to 100-fold more contig clusters for these three genes. The contigs, assembled by Xander, were longer in length and shared higher percent aa identity with known reference sequences.

In ordination analysis, these samples were grouped by crop, with corn being more distinct from the two perennial crops. This is consistent with 16S rRNA amplicon data and whole metagenomic analysis using the same samples that showed corn communities differed from those of perennial crops (Jiarong Guo, personal communication).

Using the closest matching *rplB* reference sequences, five phyla were present at 5 % or more of the total: Proteobacteria, Actinobacteria, Firmicutes, Acidobacteria, and Bacteroidetes. The distribution is similar to 16S rRNA amplicon sequence data [37] with the exception of the Acidobacteria, a typical major soil phylum. The reference set available for *rplB* is much smaller than those available for rRNA genes, and for some groups of common soil organisms there are few available reference sequences. This is especially true for the Acidobacteria and Verrucomicrobia where there are few cultured representatives. For the Acidobacteria, there are at least 26 subgroups roughly equivalent to classes [38], yet there are only 8 genome sequences, hence *rplB* sequences, available and from only 3 of these class-level subgroups. This affects the outcome of Xander at several steps: our HMM model may be biased against sequences with no relatives in the training set; if available references do not share any peptide of, in this case, 15 aa with an unknown, we will fail to identify an appropriate starting kmer; and assembled contigs may be misclassified if there are no related references available. Targeted genome sequencing efforts and single-cell sequencing have been filling in some gaps [39, 40], so this may become less of an issue.

About 10 % of bacteria were estimated to carry the *nirK* gene for the 21 samples (Table 4), similar to the qPCR estimates of 6 % *nirK* genes per rRNA gene (a multi-copy gene) in agricultural soil [41]. The *nirK* gene contigs, as expected, were dominated by matches in the Proteobacteria. However, when extrapolating from nearest gene match to taxonomic affiliation, it is important to keep in mind that some horizontal transfer of *nirK* has been detected [42]. One reference, *B. japonicum* USDA 110 (BAC52354), was the most abundant match in all three crop systems. These contigs averaged 91 % aa identity with a maximum of 98.4 % aa identity to the reference. Intriguingly, in bulk soil from Illinois sites growing the same three cropping systems, this same strain was found as having the best match to PCR amplicons targeting *nosZ*, encoding the terminal step in the denitrification pathway, although these matches were not as abundant [43]. This strain was widely used in soybean inoculants sold throughout the U.S. Midwest.

The HMP-defined community metagenomic data proved to be a good test set for validating Xander. While the community contains members from diverse taxonomic groups, several members are closely related and shared kmers of lengths 30 to 60 in their *rplB* genes, creating complexity in the graph structure. The overlap between the *rplB* gene sequences in the community combined with sequencing errors lead to small differences in the assembled sequences. The dataset was also challenging because of the low sequencing depth and regions of zero coverage of many members. Unfortunately, none of the members have *nirK* genes. Two members have *nifH* genes, but only one had all kmers of length 30 in the data*.*

We consider accuracy more important than longer and more contigs when assessing the assembler quality. For the defined community, we can measure the accuracy using the sequence identity of the recovered sequences to the known member sequences. The kmer length is an important factor in determining accuracy. A kmer length of 30 resulted in a high percentage of chimeric contig clusters. A kmer length of 45 assembled contig clusters with fewer mismatches and no chimeras but ended up with shorter contigs due to more regions with zero coverage. None of the *rplB* sequences from the defined community member are covered by kmers of length 60. For this dataset, length 45 appears to be a good choice to balance the trade-off between length and accuracy. We compared the performance of Xander to SAT-Assembler, another recently published gene-targeted assembly tool. Xander produced more and longer contigs with higher identity for more defined community members using a small dataset (Table 2). In experiments mixing the HMP community data with the corn 1 sample, SAT-Assembler did not return any non-chimeric contigs greater than 450 bp and with greater than 90 % identity to community member *rplB* genes. In contrast, Xander returned contigs corresponding to three community members.

Chimeras appeared to be a much smaller problem for contigs assembled from the rhizosphere soil data. Bulk assemblers attempt to avoid chimeras during assembly, often by terminating contigs where paths fork. In contrast, Xander relies on appropriate kmer size to reduce chimeras and post-processing to discard chimeric assemblies. On the HMP community data, UCHIME was very accurate and detected all chimeras found. We confirmed these results from UCHIME by manually validating all the contigs. It seems likely that because of the higher diversity, and potentially competitive exclusion, fewer sequences in the rhizosphere data share kmers of length 45.

Because Xander scores paths at the aa level, sequencing errors that translate into less likely amino acids for a given position will not be incorporated into a best path if an error-free read covering the same position exists; however, sequencing errors resulting in synonymous codons or resulting in more likely amino acids may be incorporated. Requiring a kmer count of 2 removed most sequencing errors and had the additional advantage of reducing the number of vertices searched by Xander. The latter is at least partially due to a reduction of starting kmers found in the graph (Table 1). For low-coverage datasets such as these, a minimum count of 2 decreased the length and number of contigs returned. For example, in sample C1, all five high-quality contigs (full-length and sharing 99 to 100 % aa identity with references) have multiple kmers of abundance 1 and so cannot be assembled with count 2. More than 35 % of the kmers in the Xander-assembled *nirK* and *rplB* contigs occurred only once in the reads (Fig. 3), while more than 40 % of *nirK* and *rplB* contigs identified in bulk assembly had median read coverage of 1 (data not shown). Xander worked well without discarding the reads with low abundance or partitioning the dataset. Such techniques are commonly employed by bulk assemblers but usually cause information loss [4].

When the search reaches a zero coverage region, meaning the gene is not complete, Xander will try to explore all available paths. It will eventually find the least bad path even though it is not related to the target gene. We used a heuristic algorithm to prune non-productive paths that reduces search space by at least tenfold with essentially identical results. This is a very simple heuristic that prunes paths that have not improved the overall path score within the user-specified number of steps. This works well for these target genes, but a more sophisticated heuristic might reduce the search space even further.

To save memory, Xander uses a Bloom filter for the in-memory representation of the de Bruijn graph [12] but is not dependent on this particular de Bruijn representation. In particular, a succinct de Bruijn graph representation has been used in some new assemblers such as MEGAHIT [44] to reduce space. Such a structure could be adopted in Xander to achieve additional space savings. Since only specific genes are targeted, pre-screening of reads could be used before the de Bruijn graph assembly to reduce space requirements even further. For example, SALT can filter out most non-target reads with minimal loss of target-gene reads by using read overlap information determined by scanning the reads through a set of relaxed HMM models [45]. Most bulk assemblers implement heuristics [46] to reduce the search space and remove errors and these could potentially be incorporated into Xander.

## Conclusions

An HMM-guided assembly using Xander combines gene assignment and assembly and allows rapid analysis of functional genes in metagenomic datasets without requiring bulk assembly or post-processing assembled data to find genes of interest. Compared to a whole metagenomic assembly where the three target genes were rarely detected, Xander assembled more contigs that were longer in length and shared higher amino acid identity with known reference sequences. Analysis of the assembly output demonstrated its ability to detect low-abundance genes and genes from low-abundance organisms, suggesting Xander can be a powerful tool to study the composition and diversity of microbial communities. HMMs used for assembly can be tailored to the targeted genes, allowing flexibility to improve annotation over generic annotation pipelines, while the ability to find multiple best paths provides Xander the potential to explore strain-level (ecotype) variation.

## Availability and requirements

**Project name:** Xander

**Project home page:**https://github.com/rdpstaff/Xander_assembler

**Operating system(s):** Platform independent

**Programming language:** Java [47]

**Other requirements:** Java 1.6 or above

**License:** GNU GPLv3

**Any restrictions to use by non-academics:** None

## Additional file

Additional file 1:
**Supplemental materials and tables about the HMP-defined community data, rhizosphere metagenomic datasets, and assembly summary results.**

## Abbreviations

CAG: combined weighted assembly graph
DG: de Bruijn graph
GB: gigabytes
Gbp: giga base pairs
HMM: hidden Markov model
kmer: substring of length k
Mbp: million base pairs
*nifH*: nitrogenase reductase gene
*nirK*: nitrite reductase gene
OTU: operational taxonomic unit
PCA: principal component analysis
*rplB*: ribosomal protein L2 gene
