# Supplementary material for: Xander: employing a novel method for efficient gene-targeted metagenomic assembly
Source: Microbiome. 2015 Aug 5;3:32. doi: 10.1186/s40168-015-0093-6 (PMC4526283; doi:10.1186/s40168-015-0093-6)
Supplement: Additional file 1: — Supplemental materials and tables about the HMP-defined community data, rhizosphere metagenomic datasets, and assembly summary results. [file 40168_2015_93_MOESM1_ESM.pdf]

Xander: Employing a Novel Method for Efficient Gene-Targeted Metagenomic Assembly

Qiong Wang, Jordan A Fish, Mariah Gilman, Yanni Sun, C Titus Brown, James M Tiedje, James R Cole

Supplemental

HMP Defined Community Data

Using the quality-filtered reads (905 Mbp; 1.7 GB file size; with an average length of 66 bp), we built a 0.5 GB de Bruijn graph with a kmer length of 30 and minimum kmer cutoff 1 (count 1 defined community graph). All the graphs used in this work had false positive rates less than 1%. We found that all kmers of length 30 making up the *rplB* sequences of defined community members *Deinococcus radiodurans*, *Acinetobacter baumannii*, *Rhodobacter sphaeroides* and *Staphylococcus epidermidis* were present in the defined community data. We searched the defined community graph using all the starting kmers identified in the reads using the 20 bacterial members of the defined community (Table S1) as reference set. From 5,044 starts we assembled 5,044 contigs and recovered 2,854 contigs of length greater than 450 bp with bit scores greater than 50. These contigs were composed of 147 different sequences, of which 36 were full-length. Of these full-length sequences, one perfectly matched *D. radiodurans* and 17 others matched *D. radiodurans* or *Staphylococcus aureus* with between one and nine mismatches. The other 18 appeared to be chimeric sequences between parents *Staphylococcus epidermidis*, *Staphylococcus aureus*, *Streptococcus mutans* and *A. baumannii.* One contig that was 15 bases shorter at the 3’ end

shared 100% nucleotide identity with *R. sphaeroides*.

Next we created a de Bruijn graph requiring a minimum kmer count of two (count 2 defined community graph). Only *D. radiodurans and Staphylococcus epidermidis* had all kmers in the count 2 graph. Repeating the search with 3,455 starting kmers assembled 3,455 contigs, with 1,178 of these having lengths greater than 450 and bit scores greater than 50. These contigs formed 17 unique sequences. Nine of these were full-length, with one a perfect match to the *D. radiodurans* and another with three base differences to *Staphylococcus aureus*. The remaining full-length sequences appeared to be chimeras between *Staphylococcus epidermidis*, *Staphylococcus aureus*, and *Streptococcus mutans*.

We expected using a larger size kmer would reduce the number of chimeras returned. Only all kmers of length 45 of *Staphylococcus epidermidis* were present in the defined community data. The kmers near the 5' end of *D. radiodurans* (up to position 192) and *Staphylococcus aureus* (up to position 120) were not present in the data. Using 2,860 starting kmers of length 45, without pruning or with prune 20, we assembled 887 contigs (nucleotide length > 450, bit score > 50) with a total of 36 unique sequences. Of these, four were full-length, one perfectly matched *Staphylococcus epidermidis*, and three had one to five mismatches to *Staphylococcus aureus*. Five partial-length contigs (477 to 675 bps) shared 99.7% to 100% nucleotide identity with *D. radiodurans*. After examining the

longest of these contigs, we found that it was truncated at the position where a kmer from *D. radiodurans* was not present in the data due to absence of reads with overlap of length 44 at this position.

We also tested if clustering contigs at 99% aa identity can group contigs together to avoid over-counting contigs because multiple start kmers that would be expected to lead to identical contigs may instead produce near-identical contigs, likely caused by forward and reverse path variations. The above 36 unique contigs formed 6 clusters. No chimeras were detected. Of the representative nucleotide contigs, one full-length perfectly matched *Staphylococcus epidermidis*, one full-length differed by 3 bases from *Streptococcus aureus*, one partial-length (675 bases) shared 100% nucleotide identity with *D. radiodurans*, and the remaining three partial-length representatives shared 96.7% to 99.7% nucleotide identity with *Streptococcus mutans* or *Streptococcus aureus.*

Figures

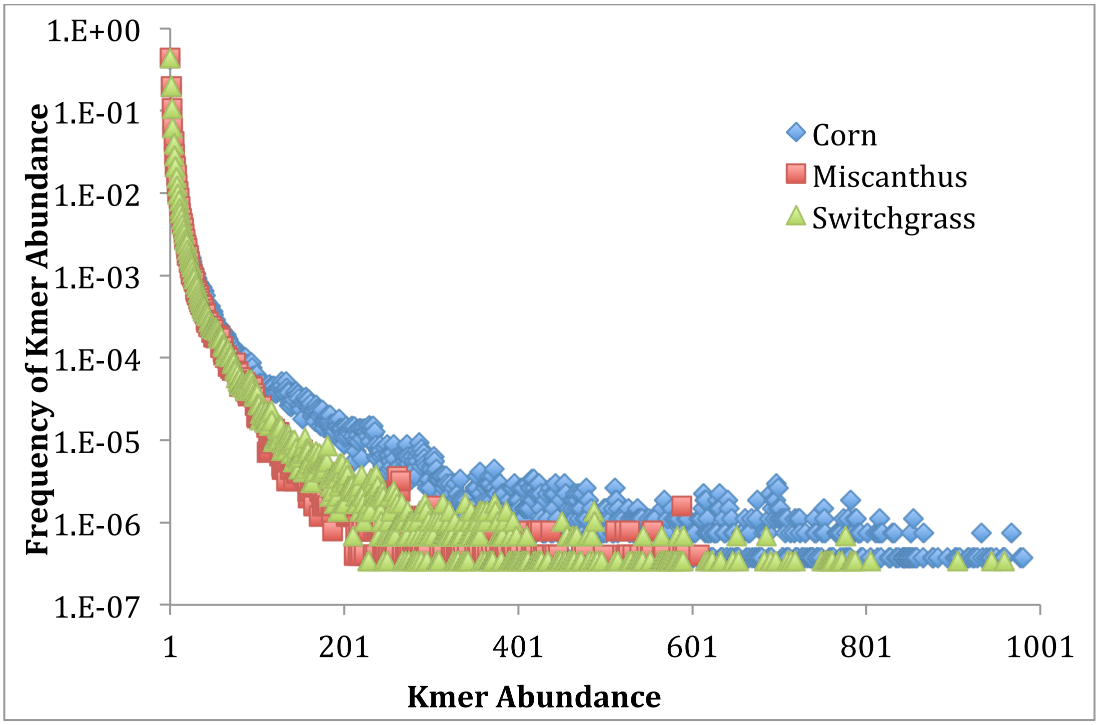

Figure S1: Kmer abundance of ribosomal protein L2 gene *(rplB)* representative contigs assembled by Xander from the pooled rhizosphere samples. The representative contigs were chosen from clusters at 99% aa identity. X-axis indicates the number of times (abundance) a kmer in the contigs occurred in the reads. Y-axis represents the fraction of total unique kmers with this abundance.

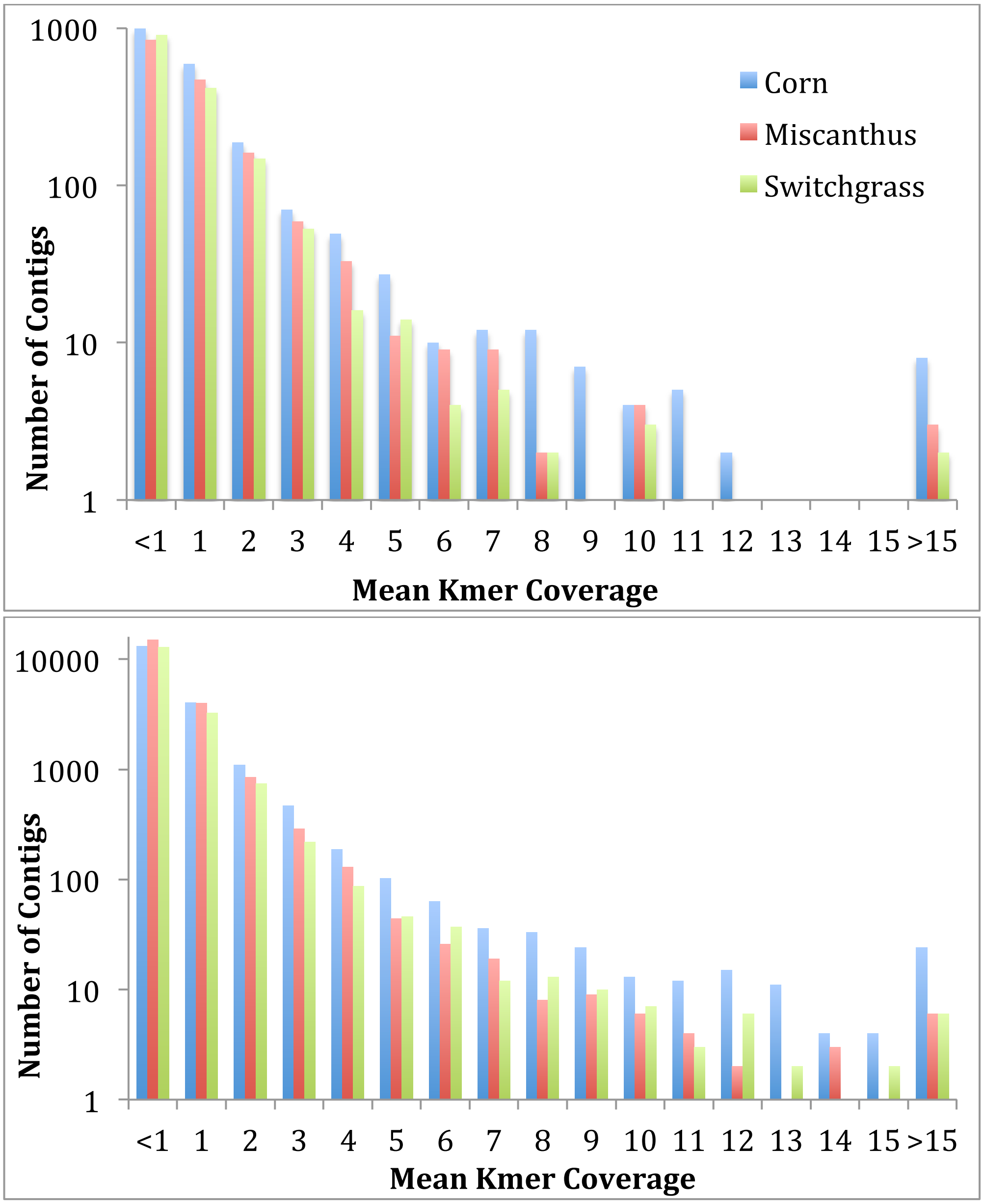

Figure S2: Mean kmer coverage of representative contigs assembled by Xander from the pooled rhizosphere samples. The representative contigs were chosen from clusters at 99% aa identity. The counts of kmers that occurred in multiple contigs were equally divided. A large portion of contigs had mean kmer coverage less than 1 likely because more than 50% kmers occurred only once or twice in the samples (Fig. 1). Top: nitrite reductase gene *(nirK).* Bottom: ribosomal protein L2 gene *(rplB).*

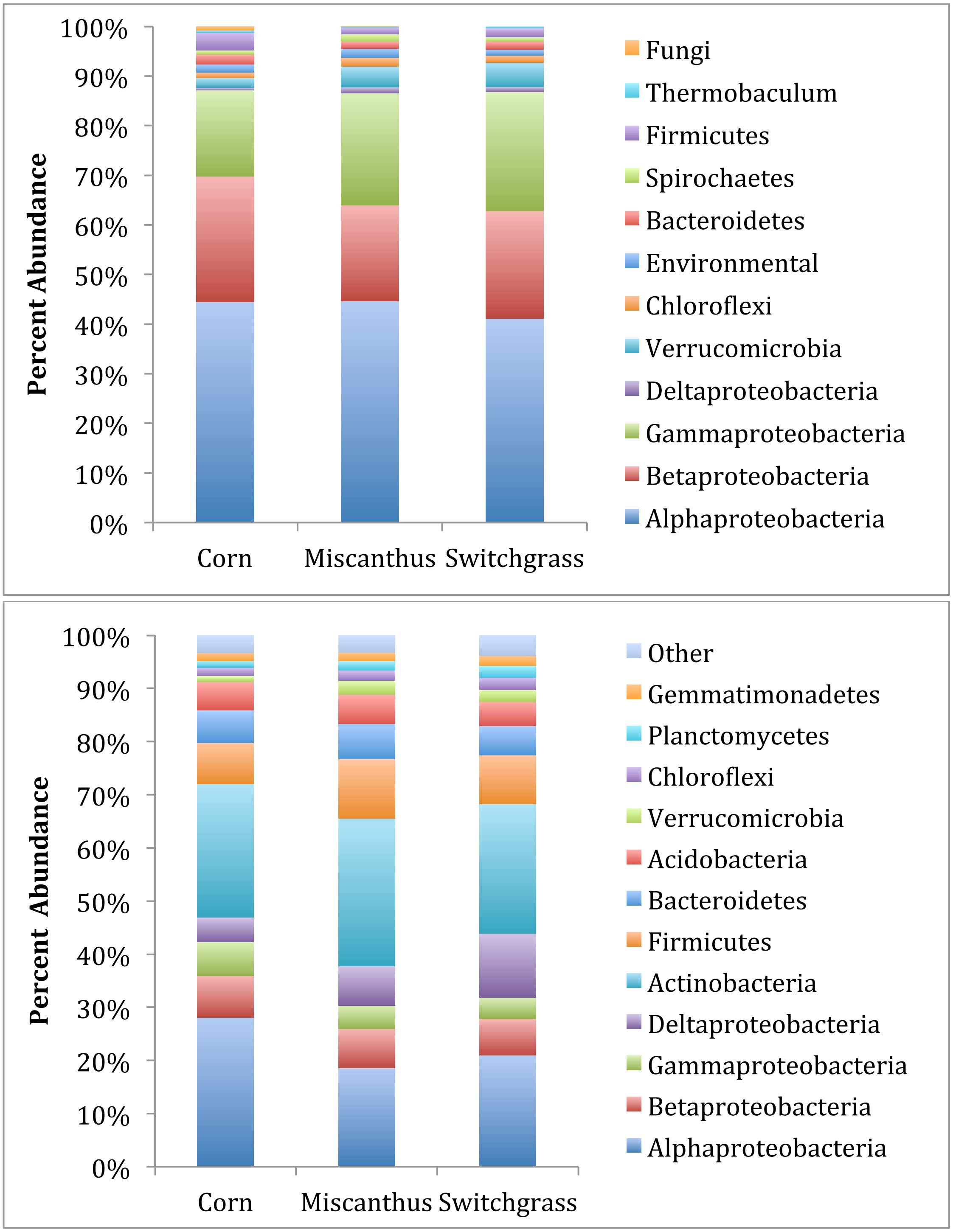

Figure S3. The taxonomic abundance based on the closest matches for the representative contigs assembled by Xander from the pooled rhizosphere samples. The representative contigs were chosen from clusters at 99% aa identity. Top: nitrite reductase *(nirK).* Bottom: ribosomal protein L2 *(rplB).*

Table S1: **HMP defined community composition**

Organism Name	Strain	Accession Number
Streptococcus mutans	NN2025 DNA	NC_013928 (AP010655)†
Listeria monocytogenes	L99 serovar 4a	NC_003210 (FM211688)†
Acinetobacter baumannii	ATCC 17978	NC_009085 (CP000521)
Actinomyces odontolyticus	ATCC 17982	DS264586.1
Bacillus cereus	ATCC 10987	AE017194.1
Bacteroides vulgatus	ATCC 8482	CP000139.1
*Candida albicans**	SC5314 assembly 21	N/A
Clostridium beijerinckii	NCIMB 8052	CP000721.1
Deinococcus radiodurans	R1 chromosome 1	AE000513.1
Enterococcus faecalis	OG1RF chromosome	CP002621.1
Escherichia coli	K12	NC_000913.2
Helicobacter pylori	26695	NC_000915.1
Lactobacillus gasseri	ATCC 33323	NC_008530.1
*Methanobrevibacter smithii**	ATCC 35061	NC_009515.1
Neisseria meningitidis	MC58	NC_003112.2
Propionibacterium acnes	KPA171202	NC_006085.1
Pseudomonas aeruginosa	PAO1	NC_002516.2
Rhodobacter sphaeroides	2.4.1 chromosome 1	NC_007493.1
Staphylococcus aureus subsp. aureus	USA300 TCH1516	NC_010079.1
Staphylococcus epidermidis	ATCC 12228	NC_004461.1
Streptococcus agalactiae	2603V/R	NC_004116.1
Streptococcus pneumoniae	TIGR4	NC_003028.3

† indicates genome records with incomplete annotations, annotations from another assembly of a synonymous strain (the accession number in parenthesis) were used instead.

* indicates genomes that were not considered in the analysis.

Table S2: Rhizosphere soil samples

Sample Name	Data Size (GB)	JGI Project ID	MG-RAST ID
C1	46	1023764	4543770.3
C2	39	1023767	4543771.3
C3	57	1023770	4543772.3
C4	53	1023773	4543773.3
C5	57	1023776	4543774.3
C6	51	1023779	4543775.3
C7	46	1023782	4543776.3
M1	56	1023785	4543777.3
M2	57	1023788	4543778.3
M3	50	1023791	4543779.3
M4	42	1023794	4543780.3
M5	45	1023797	4543781.3
M6	43	1023800	4543782.3
M7	32	1018623, 1018611	4543783.3
S1	40	1023803	4543784.3
S2	28	1018626, 1018614	4543785.3
S3	44	1023806	4543786.3
S4	49	1023809	4543787.3
S5	27	1018629, 1018617	4543788.3
S6	50	1023812	4543789.3
S7	39	1023815	4543790.3

Table S3: *nirK* genes found in Xander assembly of rhizosphere soil sample

nirK	# unique starting kmers	# protein contig clusters	Median length (aa)	Longest (aa)	Median % aa identity	Max % aa identity	# reads covering included kmers	Gene Abundance
C1	28260	326	200	360	89.39	98.29	2749	0.10
C2	18428	269	187	360	90.67	100.00	2306	0.12
C3	23931	409	210	364	88.51	97.50	3151	0.10
C4	23284	439	204	367	89.74	100.00	2602	0.10
C5	24266	391	203	380	89.69	99.10	3243	0.10
C6	26584	409	217	361	89.40	100.00	2948	0.10
C7	23085	343	196	369	90.85	98.06	3093	0.12
M1	35978	474	210	364	87.99	98.16	2692	0.11
M2	24707	460	210	361	89.01	98.40	2730	0.10
M3	21281	354	209	360	89.93	97.76	2115	0.09
M4	18667	266	153.5	360	89.93	97.84	1586	0.09
M5	19600	321	203	361	90.00	97.89	1949	0.10
M6	19868	333	204	361	90.20	97.50	1761	0.09
M7	15555	211	196	364	88.89	97.63	1180	0.10
S1	13840	247	210	361	90.07	97.50	1493	0.09
S2	14514	202	203	365	90.65	97.69	962	0.09
S3	19587	319	209	364	90.32	97.22	1785	0.09
S4	20065	342	202	363	89.47	97.50	2262	0.10
S5	13291	156	186	361	90.15	98.05	1033	0.11
S6	18571	281	181	363	89.71	97.78	2029	0.09
S7	16876	314	210	360	89.37	97.78	1541	0.09

Table S4: *rplB* genes found in Xander assembly of rhizosphere soil samples

rplB	# unique starting kmers	# protein contig clusters	Median length (aa)	Longest (aa)	Median % aa identity	Max % aa identity	# reads covering included kmers	Gene Abundance
C1	162970	3682	273	279	81.69	100	27547	1
C2	135115	2566	266	278	82.42	100	18599	1
C3	193775	4839	273	281	80.95	100	31291	1
C4	189088	4920	273	281	80.37	100	26676	1
C5	196761	4691	272	281	80.95	100	31505	1
C6	207031	4737	273	279	80.36	100	28549	1
C7	167982	3304	272	280	84.98	100	26156	1
M1	188015	5168	273	280	78.76	100	25138	1
M2	192409	5355	273	281	79.71	100	26297	1
M3	180104	4822	273	281	79.27	100	22885	1
M4	152335	3821	272	281	79.64	100	17888	1
M5	150985	3981	272	281	79.27	100	20047	1
M6	168087	4385	272	279	78.26	100	19228	1
M7	122197	2732	255.5	278	80.00	100	12136	1
S1	150644	3785	271	279	80.28	100	16726	1
S2	112337	2304	227	278	81.60	100	10182	1
S3	158127	4201	272	279	79.13	100	19422	1
S4	160463	4258	272	279	79.64	100	21752	1
S5	90606	1876	218	281	80.66	100	9423	1
S6	157137	4040	272	279	78.57	100	22537	1
S7	145770	3643	272	281	79.49	100	17152	1

Table S5: *nifH* genes found in Xander assembly of rhizosphere soil samples

nifH	# unique starting kmers	# protein contig clusters	Median length (aa)	Longest (aa)	Median % aa identity	Max % aa identity	# reads covering included kmers	Gene Abundance
C1	1293	6	112	205	95.79	99.51	34	0.001
C2	1543	5	122	137	95.90	99.17	20	0.001
C3	2231	7	131	148	95.73	100.00	26	0.001
C4	2486	12	139.5	296	93.67	99.18	73	0.003
C5	2653	3	122	294	97.52	98.98	27	0.001
C6	2529	4	181.5	196	92.03	96.00	26	0.001
C7	1640	0	0	0	0.00	0.00	0	0.000
M1	3088	8	112	178	97.30	98.26	33	0.001
M2	2997	11	142	276	93.75	98.54	104	0.004
M3	2921	7	142	205	92.73	100.00	47	0.002
M4	2609	8	124.5	189	94.99	99.12	51	0.003
M5	2429	9	123	148	96.11	97.50	30	0.001
M6	2141	4	117	129	99.23	100.00	21	0.001
M7	2577	8	134.5	239	92.53	95.46	30	0.002
S1	2593	7	271	292	92.41	97.91	62	0.004
S2	1966	7	204	233	95.61	98.04	41	0.004
S3	2146	9	157	290	95.97	97.22	44	0.002
S4	1969	13	164	276	94.55	97.83	60	0.003
S5	950	0	0	0	0.00	0.00	0	0.000
S6	3035	10	195.5	295	92.61	98.17	54	0.002
S7	2334	5	217	291	92.06	97.54	29	0.002
